# Correction to: Preservation of Residual Kidney Function in the Management of Patients on Incident Hemodialysis: An Opportunity to Improve Outcomes

**DOI:** 10.34067/KID.0000000913

**Published:** 2025-07-08

**Authors:** Jochen G. Raimann, Yoshitsugu Obi

In the editorial by Raimann and Obi, “Preservation of Residual Kidney Function in the Management of Patients on Incident Hemodialysis: An Opportunity to Improve Outcomes” (*Kidney360* 2025;6(1):12–14. doi:10.34067/KID.0000000649), corrections are needed.

The authors note that this work was supported by the National Institute of General Medical Sciences of the National Institutes of Health under Award Number U54GM115428.

